# Contributions of TOR Signaling on Photosynthesis

**DOI:** 10.3390/ijms22168959

**Published:** 2021-08-20

**Authors:** Yun Song, Mohammed Salem Alyafei, Khaled Masmoudi, Abdul Jaleel, Maozhi Ren

**Affiliations:** 1 School of Life Sciences, Liaocheng University, Liaocheng 252000, China; songyun@lcu.edu.cn; 2 Department of Integrative Agriculture, College of Food and Agriculture, United Arab Emirates University, Al Ain 15551, United Arab Emirates; mohammed.s@uaeu.ac.ae (M.S.A.); khaledmasmoudi@uaeu.ac.ae (K.M.); abdul.jaleel@uaeu.ac.ae (A.J.); 3 Institute of Urban Agriculture, Chinese Academy of Agricultural Sciences, Chengdu 610213, China

**Keywords:** target of rapamycin (TOR), plant, photosynthesis, photoautotrophy, C nutrient

## Abstract

The target of rapamycin (TOR) protein kinase is an atypical Ser/Thr protein kinase and evolutionally conserved among yeasts, plants, and mammals. TOR has been established as a central hub for integrating nutrient, energy, hormone, and environmental signals in all the eukaryotes. Despite the conserved functions across eukaryotes, recent research has shed light on the multifaceted roles of TOR signaling in plant-specific functional and mechanistic features. One of the most specific features is the involvement of TOR in plant photosynthesis. The recent development of tools for the functional analysis of plant TOR has helped to uncover the involvement of TOR signaling in several steps preceding photoautotrophy and maintenance of photosynthesis. Here, we present recent novel findings relating to TOR signaling and its roles in regulating plant photosynthesis, including carbon nutrient sense, light absorptions, and leaf and chloroplast development. We also provide some gaps in our understanding of TOR function in photosynthesis that need to be addressed in the future.

## 1. The TOR Complex in Plants

During the long-term evolution from the Last Eukaryotic Common Ancestor (LECA), plants have diverged from animals and other eukaryotes in many ways. Their most prominent feature is the ability to using light energy to generate organic molecules (e.g., glucose (C_6_H_12_O_6_), sugars, starch) from carbon dioxide (CO_2_) and water (H_2_O) and release molecular oxygen (O_2_) into the atmosphere, which is also called photosynthesis [1]. Photosynthesis is an essential process and facilitates the expansion of plants broadly across all of the Earth’s biomes. With limited agricultural land and increasing human population, it is essential to decipher the key regulators involved in photosynthesis and enhance photosynthetic activities [1,2,3,4].

Living organisms possess many mechanisms to sense when nutrient availability and environmental cues are conducive to growth and development [5]. Through identification of different external or internal cues, organisms adjust their metabolism, growth, and development to survive harsh conditions. The target of rapamycin (TOR) kinase signaling cascade is a core component of nutrient sensing in all eukaryotic organisms. Recently, McCready et al. explained the importance of TOR kinase in plant development as a whole [6]. In addition, the role of the TOR signaling plants’ defense against pathogen and related metabolisms was studied recently [7]. When eukaryotes are in favorable conditions, TOR signaling pathway is active, which promotes anabolic processes and inhibits catabolism and protein degradation, but when conditions are unfavorable for growth, TOR is inactivated and promotes catabolic processes [8,9]. Xiong and Sheen explained the glucose-TOR signaling in the transcriptional control of the cell cycle and enumerated the importance of leaf photosynthesis-derived glucose in activating TOR [10].

Tremendous progress has been made in uncovering the molecular and cellular functions of TOR in regulating plant photosynthesis. In this paper, we first present a broad overview of recent developments in the plant TOR complex. Secondly, we highlight the impact of TOR signaling on plant photosynthesis, focusing on findings on the regulation of TOR in sensing C nutrient derived from photosynthesis, light absorptions, and leaf and chloroplast development. Finally, we present some pressing questions to be addressed as we move onward in the study of TOR signaling in plant photosynthesis.

**Figure 1:**
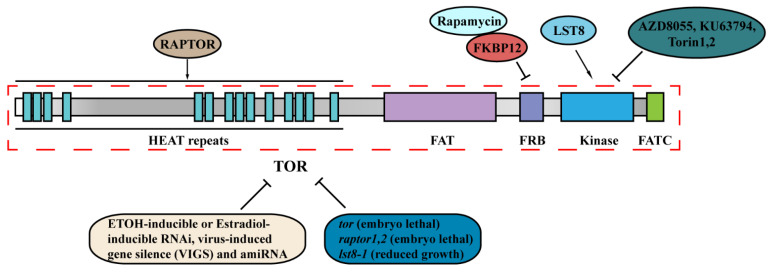
Structure of TOR protein and strategies to dissect TOR function. The TOR protein contains five conserved domains including HEAT repeats, FAT domain, FRB domain, kinase domain and FATC domain. The multifaceted functions of TOR kinase are dissected by integrated analysis of chemical inhibitor treatments, TOR RNAi seedlings and TOR signaling-related mutants. Abbreviations: TOR, Target of Rapamycin; HEAT repeats, Huntingtin, Elongation factor 3, subunit of protein phosphatase 2A and TOR1; FAT, FRAP, ATM and TRRAP domain; FRB, FKP12-rapamycin binding domain; FATC, Carboxy-terminal FAT domain; RAPTOR, regulatory-associated protein of mTOR; LST8, lethal with Sec13 protein 8; FKBP12, FK506-binding protein 12; RNAi, RNA interference; amiRNA, artificial microRNA.

Rapamycin (RAP) is an immunosuppressive macrolide antibiotic produced by the soil bacterium *Streptomyces hygroscopicus* on Easter Island, which stops yeast division and produces cells with an N starvation phenotype [11]. Through screening for genetic mutants resistant to rapamycin, FK506-binding protein 12 (FKBP12) together with TOR were first identified, and TOR emerged as a central regulatory hub in all eukaryotic organisms, including yeasts, animals, and plants [12,13,14,15,16]. TOR is a conserved serine/threonine-protein kinase and belongs to the phosphatidylinositol 3-kinase-related kinase family and has been found in all sequenced plant and algal genomes [11,17,18]. The TOR protein contains five conserved domains, including HEAT (Huntington, Elongation Factor 3 regulatory, subunit A of PP2A, TOR1) repeats, FAT (FRAP-ATM-TTRAP) domain, FRB (FKBP rapamycin-binding) domain, kinase domain and FATC domain (Figure 1). The HEAT repeats mediate protein–protein interactions and membrane associations; the FAT domain, together with the FATC domains, contributes to protein interactions and kinase activation; the FRB domain is targeted by the inhibitory FKBP12–rapamycin complex [19].

In yeast and mammalian systems, TOR exists in two structurally and functionally distinct protein complexes: the rapamycin-sensitive TOR complex 1 (TORC1) and the rapamycin-insensitive TOR complex 2 (TORC2) [20,21]. The core components of TORC1 include TOR, regulatory-associated protein of mTOR (RAPTOR)/KOG1, and lethal with SEC13 protein 8 (LST8), while TORC2 includes TOR, SAPK-interacting protein 1 (SIN1)/AVO1, rapamycin-insensitive companion of mTOR (RICTOR)/AVO3, and LST8. In mammalian systems, they have only one *TOR* gene. In yeast (*Saccharomyces cerevisiae*), two *TOR* genes, *TOR1* and *TOR2,* were identified. Either TOR1 or TOR2 can form the TORC1 complex, while only TOR2 can form the TORC2 complex [8].

Research about TOR signaling in plants lags behind that in yeast and mammals. In plant systems examined to date, most of them have only one *TOR* gene; two *TOR* genes are identified in polyploids *Glycine max*, *Populus trichocarpa*, and *Brassica rapa* [17,22]. Four *TOR* genes are identified in allotetraploid cotton *Gossypium hirsutum* [23]. These results suggest that different plant species might have evolved unique TOR complexes with specific functions. Intriguingly, TORC2 complex components SIN1 and RICTOR appear to be absent in the plant lineage, indicating that plants may not form a conserved TORC2 [8,24].

Scientists revealed that plants were insensitive to rapamycin and could not form a complex with RAP, a small 12 kDa protein FKBP12 and the FRB domain of TOR [13,21,25]. However, the growth of *Arabidopsis* seedlings or cells in liquid culture was inhibited by RAP [26,27]. To elucidate the function of AtTOR in plants’ various cellular and developmental processes, rapamycin-sensitive transgenic *A*. *thaliana* lines (BP12) expressing yeast FK506 Binding Protein12 were developed and used [28,29,30,31,32,33]. In addition, a new generation of ATP-competitive chemical inhibitors specific to TOR kinase, such as AZD8055, Torin1, Torin2, and KU0063794, have been applied for TOR studies in plants [28,32,33].

In the model plant *Arabidopsis thaliana*, one copy of *TOR* gene, two copies of *Raptor* (*RaptorA* and *RaptorB*) genes, and two copies of *LST8* (*LST8-1* and *LST8-2*) genes exist [9,17]. Studies suggested that the null *tor* mutant is embryo-lethal, the *raptora/b* double mutant is unable to maintain post-embryonic meristem-driven growth, and the *lst8-1* mutant exhibits modest dwarf growth and early senescence phenotypes [9,34,35,36]. These results revealed that *Arabidopsis* TOR, Raptor, and LST8 all play a pivotal role in regulating multiple plant growth and development, and TOR appears to regulate a much broader spectrum of biological functions than Raptor or LST8.

## 2. TOR: A Key Regulator in Photosynthesis

**Figure 2:**
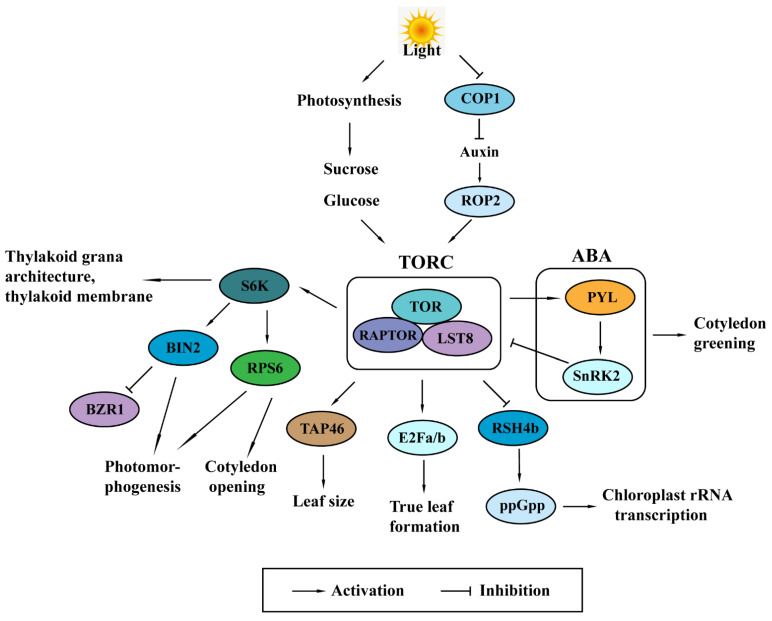
Summary of Target of Rapamycin (TOR) signaling in plant photosynthesis, including inputs for the activation of TOR signaling and output processes regulated by the TOR signaling. Light signals and sugars derived from photosynthesis activate TOR signaling and the conserved downstream outputs. The activated TOR downstream outputs regulate photomorphogenesis and leaf development, which eventually influences the efficiency of oxygenic photosynthesis. Abbreviations: COP1, constitutive photomorphogenesis 1; ROP2, Rho-like small GTPase 2; TORC, Target of Rapamycin complex; TOR, Target of Rapamycin; RAPTOR, regulatory-associated protein of mTOR; LST8, lethal with Sec13 protein 8; S6K, S6 kinase; BIN2, brassinosteroid-insensitive 2; BZR1, brassinazole resistance 1; RPS6, ribosomal protein S6; TAP46, type 2A-phosphatase-associated protein 46 kDa; E2Fa/b, E2 promoter-binding factor a/b; RSH4b, RelA-SpoT homolog gene 4b; ppGpp, guanosine 3′-diphosphate 5′-diphosphate; PYL, pyrabactin resistance 1-like; SnRK2, SNF1-related protein kinase 2.

Extensive studies have demonstrated that TOR senses and integrates nutrient, hormone, light, energy, and other environmental cues to orchestrate growth and development. As a serine-threonine protein kinase, TOR mediates changes in protein phosphorylation and modulates a myriad of important cellular and metabolic processes, including nutrient assimilation, nutrient transport, ribosome biogenesis, cell division, cell expansion, polysome integrity, metabolism, autophagy, and development [14,17,31,37,38,39]. Photosynthesis functions as one of the most important anabolic processes in plants. Recent research suggest that TOR plays an indispensable role in plant photosynthesis [40]. Plants with TOR dysfunctions showed severe defects in chloroplasts and photosynthesis [23,36,41,42,43,44]. Transcriptome analysis in TOR-inhibited plants revealed a broad regulation of plant photosynthesis-related genes [28,30,41,45]. Intriguingly, activation of TOR leading to defects in chloroplast development compared with wild type, further supports the pivotal roles of TOR in regulating plant photosynthesis [46,47]. In the present review, we summarize recent advances that address the functions of TOR in regulating plant photoautotrophy and maintenance of photosynthesis (Figure 2).

### 2.1. Role of TOR in Sensing C Nutrient

Unlike yeast and animals, plants acquired the ability to live outside water, and their most prominent feature is probably their ability to synthesize organic carbon (C) molecules like glucose, sugars (fructose, glucose or sucrose) and starch by using light energy and releasing molecular oxygen into the atmosphere, which is also called photosynthesis. The ability to sense, assimilate, transport, and utilize C nutrients is vital for plant survival and growth. Plants decide whether to store sugars in source leaves or distribute these metabolites to sink organs, such as young organs, roots, or seeds to ensure the supply-demand balance of sugar in plants [48]. Evidence supports that TOR is involved in plant C nutrient sensing and serves as an intermediate for the regulation of plant growth by organic carbon (C) sugars [9,39].

At the photoautotrophic transition checkpoint, 15 mM glucose and sucrose can promote TOR signaling and control root meristem, root elongation, cotyledon expansion, and root hair production activation through glycolysis and mitochondrial bioenergetic. The requirement for glycolysis and mitochondrial bioenergetics to activate TOR protein kinase was monitored by the phosphorylation of its conserved substrates S6K. In this process, other sugars, such as fructose, galactose, and xylose, are much less effective [27,44]. Recent findings have illustrated that glucose is sufficient to activate TOR kinase in root apexes, whereas glucose and light signals are required for TOR activation in shoot apexes. In shoot apexes, exogenously auxin can replace light to activate TOR and promote leaf development. The small GTPase Rho-related protein 2 (ROP2) transduced light-auxin signal to activate TOR and promotes TOR-dependent phosphorylation and activation of transcription factor E2Fa/E2Fb, which eventually enhances root growth and leaf formation [27,44,49].

Glucose-activated TOR also controls the accumulation of the brassinosteroid (BR)-signaling transcription factor BZR1 (Brassinazole resistance 1). Inhibition of TOR by inducible RNAi reduced expression of BR-responsive genes and abolished sugar-promoted BZR1 accumulation. The growth inhibition caused by TOR inactivation can be partially recovered by exogenously applied BR and the BZR1 gain-of-function mutation *bzr1-1D*. The regulation of BZR1 accumulation by glucose-TOR allows carbon availability to control plant growth [50].

Through genetic approaches and chemical or physiological treatments to either promote or disrupt TOR activity, the results indicated that TOR restricted the plasmodesmata (PD) transport activity in leaves. Further study revealed that TOR was significantly more active in mature leaves photosynthesizing excess sugars (glucose) than in young and growing leaves [51].

In addition to glucose, sucrose is demonstrated to play a pivotal role in TOR activation and development of shoot apical meristem. Research suggested that sucrose was essential to promote leaf expansion even in the *cop1* mutants, which exhibited constitutive photomorphogenesis with open cotyledons and a short hypocotyl. TOR protein plays a key role in integrating energy and light signaling to promote stem cell activation in the shoot apical meristem [52].

### 2.2. Light and TOR Signaling Pathway

Plants are oxygenic photosynthesizers that use light energy to generate organic molecules from carbon dioxide (CO_2_) and water (H_2_O). The ability to absorb the light energy has a significant effect on the photosynthetic efficiency of plants. In addition, light-stimulated photomorphogenesis is a developmental process that transforms plants into a vegetative state, which is required for photosynthetic activity. Light regulates the expression pattern of genes that participated in cell proliferation, organ development, and differentiation of photosynthetic cells. Advancing research has shown that light is intimately linked with TOR activation.

In *Arabidopsis* shoot apexes, both light signals and the glucose are indispensable for continuously promoting auxin accumulation for TOR activation to facilitate conversion to photoautotropic growth in *Arabidopsis*. Light-auxin signal can activate ROP2, which then promotes TOR kinase activity [49]. In deetiolating *Arabidopsis* seedlings, phytochrome A and cryptochromes perceive light to inactivate the negative regulator Constitutive photomorphogenesis 1 (COP1), and then leads to TOR activation and phosphorylation of downstream protein RPS6. The activation of TOR and RPS6 enhances cotyledon opening and transformation from young plant seedlings into the vegetative phase with photosynthetic activities [53]. This finding reveals the molecular mechanism of TOR activation in light-specific translational enhancement in plants de-etiolation.

In addition, the sugar requirement for TOR–S6K–RPS6 activation was investigated in older etiolated seedlings. RPS6 phosphorylation was elevated by light exposure for 2 h, but not for 0.5 h. However, when seedlings were grown with 1% sucrose, RPS6 phosphorylation was promoted by light as short as 0.5 h [54]. A recent study demonstrated that the impairment of TOR activity (through RAPTOR1B mutations or TOR kinase active site chemical inhibition) in *Arabidopsis*, reduced the accumulation of the photoreactive chlorophyll precursor protochlorophyllide in darkness, but increased greening rate of etiolated seedlings after exposure to light [55].

### 2.3. TOR Regulates Leaf and Chloroplast Development

Plant chloroplasts are essential organelles that carry out oxygenic photosynthesis. Several studies revealed the involvement of TOR signaling in the biogenesis and maturation of chloroplasts [23,29,33,45,48,55,56]. The suppression of TOR by active-site TOR inhibitor AZD8055 in *Arabidopsis* eliminated greening and cotyledon expansion and remodeled the expression profile of genes associated with photosynthesis, involved in chlorophyll biosynthesis, light reactions, and CO_2_ fixation [45]. The transcription and translation of nuclear-encoded mRNAs coding for plastidic ribosomal proteins were down-regulated in TOR inactivation plants (ethanol-inducible TOR RNAi lines), which could explain the chlorotic phenotype of the TOR silenced plants [48]. Recent findings also uncovered the genes involved in plastid biogenesis were particularly sensitive to TOR inhibition by AZD8055 treatment [57]. Analysis of the *C. reinhardtii* proteome and phosphoproteome under TOR inactivation revealed that the levels of most of the proteins related to the Calvin cycle were decreased, suggesting the involvement of TOR in photosynthesis [58].

Inhibition of TOR activity resulted in reduced chloroplast number and size, and thus impaired photoautotrophic growth in *Arabidopsis* [33]. In addition, scientists identified the brassinosteroid insensitive 2 (BIN2) as a novel phosphorylation target of TOR-S6K2 and uncovered the pivotal role of TOR-S6K2-BIN2 in heterotrophic to photoautotrophic transition [33]. In addition, TOR inhibition caused altered chloroplast morphology, increased non-photochemical quenching (NPQ), damage to photosystem II (PS II) reaction centers and inhibited efficient state transitions between PSII and PSI in alga [59]. In allotetraploid cotton *Gossypium hirsutum*, pharmacological experiments with TOR inhibitor AZD8055 and silencing *GhTOR* genes by virus-induced gene silencing (VIGS) resulted in a significant delay in the transition from heterotrophic to photoautotrophic growth [23].

Ectopic expression of full-length AtTOR increased plant photosynthetic efficiency and chlorophyll content in rice [60]. Type 2A phosphatase-associated protein of 46 kDa (TAP46), a regulatory subunit of protein phosphatase 2A, is a direct TORC1 phosphorylation substrate. Transgenic plants overexpressing TAP46 exhibited increased hypocotyl length and enlarged leaves [61,62]. Ribosomal protein S6 kinase (S6K) functions as a key component in the target of rapamycin (TOR) pathway. In rice (*Oryza sativa L.*), suppression of S6K1 caused pale yellow-green leaves and defective thylakoid grana architecture. Genetic and pharmacological evidence further demonstrated that S6K1 was vital for galactolipid biosynthesis for the thylakoid membrane [56]. Arabidopsis *raptor1b* mutation resulted in decreased CO_2_ assimilation rate and increased stomatal conductance; however, the chloroplast development or photosynthetic electron transport efficiency was not affected [42].

In *Arabidopsis*, TOR functioned as a positive regulator of chlorophyll biosynthesis and metabolism via TRIN1 (*tor-inhibitor insensitive 1*)/ABI4 (abscisic acid-insensitive 4) at the photoautotrophic stage. The precise molecular connection between TOR and ABI4 remains to be elucidated [29]. The TOR complex components *lst8* and *raptor* mutants as well as wild-type *Arabidopsis* seedlings under TOR inhibition by AZD showed diminished ABA hormone accumulation. The diminished ABA hormone accumulation was correlated with decreased transcript levels of ABA biosynthetic enzymes including zeaxanthin epoxidase (ZEP), the 9-cis-epoxycarotenoid dioxygenase NCED3 and aldehyde oxidases AAO3, and increased transcript amount of the abscisic acid catabolism gene cytochrome P450 monooxygenases CYP707A2 [63]. Furthermore, an in vitro protein kinase screen identified that TOR kinase phosphorylated ABA receptors PYL at a conserved serine residue S119 [64]. These results suggested the reciprocal regulations between TOR and ABA signaling to balance plant growth and stress responses, consistent with the role of TOR and ABA in seedling development and greening of cotyledons and leaves [39].

Scientists revealed that inhibition of TOR increased the expression level of a nuclear-encoded chloroplast RelA-SpoT homolog (RSH) gene *CmRSH4b*. *CmRSH4b* encoded the guanosine 3′-diphosphate 5′-diphosphate (ppGpp) synthetases and regulated chloroplast rRNA transcription in the unicellular red alga *Cyanidioschyzon merolae* [65]. Researchers examined changes in oxidation during TOR inhibition in green alga *Chlamydomonas reinhardtii*. A reduction in photosynthetic electron flow and twenty proteins related with photosynthesis were identified with altered oxidation status under TOR inhibition [66].

## 3. Conclusions and Future Prospects

The analysis of the recent research progress and key findings on the TOR signaling pathways in plant photosynthesis suggests that TOR is a crucial signal integrator to transduce nutrient and environmental inputs into physiological, molecular, and developmental responses for growth. TOR kinase senses C nutrients and is activated by the availability of sugar produced by light-stimulated photosynthesis. Active TOR protein enhances plant photomorphogenesis and leaf development to make sure the maintenance of photosynthesis.

Compared with measurable progress made in the TOR signaling of mammalians and yeasts, our understanding of plant and algal TOR is limited. Although these findings in the present study clearly highlight a role for TOR in plant photosynthesis, several questions remain to be answered. Do plants possess unique TOR complex involved in plant specific life process photosynthesis? How do plants sense upstream signals for sugars and light? Plant hormones auxin, ABA and BR are involved in the regulation of TOR signaling and photosynthesis. What is the molecular mechanism of these hormones on TOR? How does TOR integrated into the known canonical hormone signaling? Are other hormones such as salicylic acid, jasmonic acid and ethylene involved in TOR regulation? In addition, these findings highlight a role for TOR in leaf and chloroplast development. What are the downstream effectors that mediate TOR function in this context?

In future studies, it is necessary to decipher the underpinning molecular mechanism of TOR in regulating plant photosynthesis. Genetic and biochemical analyses combined with currently available and future data from phosphoproteomic, transcriptomic and metabolomic are effective approaches to identify TOR functions and discover TOR up- and down-stream effectors.

With limited agricultural land and increasing human population, it is essential to enhance crop productivity. Increasing the photosynthesis rate of plants has been recently revitalized as one of the approaches for increasing grain crop yields and solving world food crises [1,2,3,4]. Understanding the regulation of TOR signaling in plants photosynthesis may be essential for crop improvement in the future.

## Data Availability

Not applicable.
